# The molecular mechanism of phytosphingosine binding to FFAR4/GPR120 differs from that of other fatty acids

**DOI:** 10.1002/2211-5463.13301

**Published:** 2021-10-03

**Authors:** Tomotaka Nagasawa, Masaki Horitani, Shin‐ichi Kawaguchi, Shigeki Higashiyama, Yoichiro Hama, Susumu Mitsutake

**Affiliations:** ^1^ The United Graduate School of Agricultural Sciences Kagoshima University Kagoshima Japan; ^2^ Department of Applied Biochemistry and Food Science Faculty of Agriculture Saga University Saga Japan; ^3^ Center for Education and Research in Agricultural Innovation Faculty of Agriculture Saga University Saga Japan; ^4^ Division of Cell Growth and Tumor Regulation Proteo‐Science Center Ehime University Matsuyama Japan; ^5^ Department of Molecular and Cellular Biology Osaka International Cancer Institute Osaka Japan

**Keywords:** FFAR4, GPCR, GPR120, phytosphingosine, sphingolipid, sphingosine

## Abstract

Free fatty acid receptor 4 (FFAR4)/GPR120 comprises a receptor for medium‐ and long‐chain fatty acids. We previously identified phytosphingosine (PHS) as a novel ligand of FFAR4. Although many natural FFAR4 ligands have carboxyl groups, PHS does not, thus suggesting that binding to FFAR4 is driven by a completely different mechanism than other natural ligands such as α‐linolenic acid (ALA). To test this hypothesis, we performed docking simulation analysis using a FFAR4 homology model based on a protein model derived from the crystal structure of activated turkey beta‐1 adrenoceptor. The docking simulation revealed that the probable hydrogen bonds to FFAR4 differ between various ligands. In particular, binding was predicted between R264 of the FFAR4 and the oxygen of the carboxylate group in ALA, as well as between E249 of the FFAR4 and the oxygen of the hydroxy group at the C4‐position in PHS. Alanine substitution at E249 (E249A) dramatically reduced PHS‐induced FFAR4 activation but demonstrated a weaker effect on ALA‐induced FFAR4 activation. Kinetic analysis and *K*_m_ values clearly demonstrated that the E249A substitution resulted in reduced affinity for PHS but not for ALA. Additionally, we observed that sphingosine, lacking a hydroxyl group at C4‐position, could not activate FFAR4. Our data show that E249 of the FFAR4 receptor is crucial for binding to the hydroxy group at the C4‐position in PHS, and this is a completely different molecular mechanism of binding from ALA. Because GPR120 agonists have attracted attention as treatments for type 2 diabetes, our findings may provide new insights into their development.

## Graphical abstract

Free fatty acid receptor 4 (FFAR4)/GPR120 is known as a receptor for medium‐ and long‐chain fatty acids. We previously identified phytosphingosine (PHS) as a novel ligand of FFAR4. The present study demonstrated that E249 of the FFAR4 receptor is crucial for binding to the hydroxy group at the C4‐position in PHS, and this is a completely different molecular mechanism of binding from α‐linolenic acid.

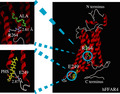

**Abbreviations**
ALA: α‐linolenic acid
AP: alkaline phosphatase
FFAR1: free fatty acid receptor 1
FFAR4: free fatty acid receptor 4
GLP‐1: glucagon‐like peptide‐1
GPCR: G protein‐coupled receptor
PDB: Protein Data Bank
PHS: phytosphingosine
SPH: sphingosine
TGFα: transforming growth factor‐α
TM: transmembrane
β_1_AR: beta‐1 adrenoceptor

Free fatty acid receptor 4 (FFAR4)/GPR120 comprises a receptor for medium‐ and long‐chain fatty acids that is expressed in small intestinal endocrine cells, L cells and adipose tissue. Activation of FFAR4 promotes the secretion of glucagon‐like peptide‐1 (GLP‐1) [1], which is known as an intestinal hormone incretin. GLP‐1 was reported to suppress appetite and increase insulin secretion, exhibiting anti‐diabetic effects [2, 3, 4]. Dipeptidyl peptidase‐4, which degrades GLP‐1, is known to extend the half‐life of GLP‐1 in blood and enhance its anti‐diabetic action. Inhibitors of this enzyme have already developed as novel molecular targeted pharmaceuticals for type 2 diabetes [5]. Long‐chain fatty acid/FFAR4 signaling, which is responsible for incretin secretion, is an attractive pharmaceutical target for the treatment of type 2 diabetes. Thus, extensive screening studies for new ligands of FFAR4 have been conducted. Many FFAR4 ligands have been reported, including natural ligands. Many natural ligands and synthetic ligands possess carboxyl groups [1, 6]. In a previous study, we developed a new method for screening FFAR4 ligands, and identified phytosphingosine (PHS) as a novel ligand [7]. PHS is present at high levels in yeast, and it is a component of the plasma membrane. Interestingly, PHS has no carboxyl groups, suggesting that it interacts with FFAR4 in a different manner than natural ligands possessing carboxyl groups.

Recent research efforts revealed the crystal structures and activation mechanisms of G protein‐coupled receptors (GPCRs) [8]. Although an increasing numbers of reports are available for the fatty acid receptor, GPR40 (FFAR1) [9, 10, 11], few studies have described modeled structures of FFAR4, hindering our understanding of its physiological functions. In the presennt study, we established a FFAR4 homology model and docked the receptor with PHS and α‐linolenic acid (ALA) to reveal their different mechanisms of action.

## Materials and methods

### Homology modeling

A search using the blast alignment algorithm (https://blast.ncbi.nlm.nih.gov/Blast.cgi) within the Protein Data Bank database (PDB) (https://www.rcsb.org) revealed various potential templates for molecular modeling [12, 13]. Crystal structures exhibited high identity scores and maximum query coverage with respect to FFAR4. The crystal structure of activated turkey beta‐1 adrenoceptor (β_1_AR) (PDB ID: 6IBL) [14] was used as a template to build the structure of FFAR4. The 3D models of FFAR4 were built using modeller, version 9.25 [15, 16].

### Docking simulation of PHS using a FFAR4 homology model

**Fig. 1:**
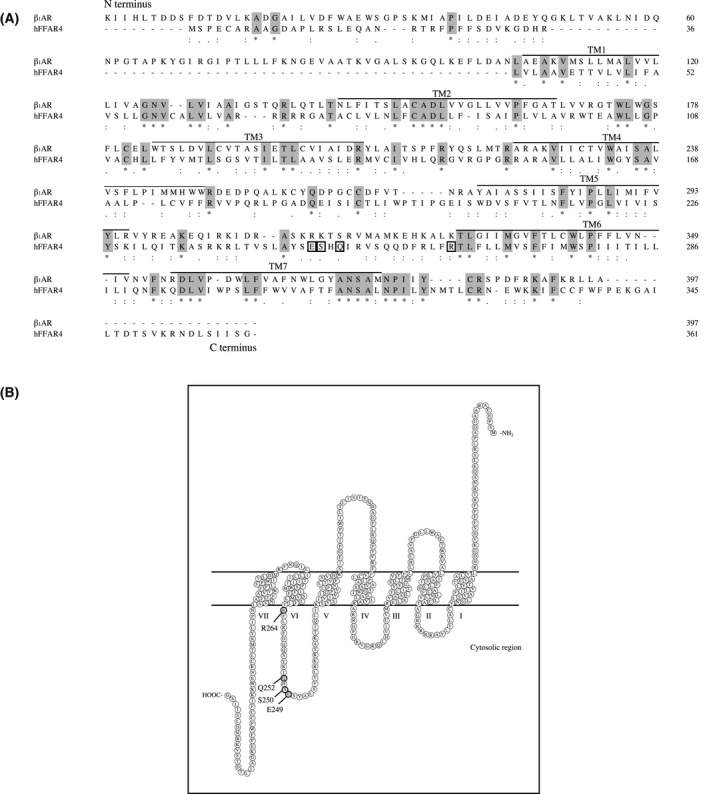
Amino acid sequence alignment between activated turkey β_1_AR and FFAR4. (A) Amino acid sequences corresponding to activated β_1_AR (PDB ID: 6IBL) and human FFAR4/GPR120 (GenBank accession no. BC101175) were aligned using clustalw [16]. TM1–7, seven TM domains; * (gray space), residues of β_1_AR and hFFAR4 in the sequence alignment are identical; :, conserved residues observed; •, semiconserved residues observed; square black frame, the possible interacting residues. (B) The predicted Snake‐like plot of the hFFAR4 was built using sosui [20].

Phytosphingosine was built systematically using chemsketch and pymol (DeLano Scientific, San Carlos, CA, USA). ALA was obtained as a ligand from PDB. We used the crystal structure of activated β_1_AR to develop a homology model of FFAR4 [17, 18]. The sequence alignment of β_1_AR [19] and FFAR4 is shown in Fig. 1. The FFAR4 model featured seven transmembrane (TM) domains, in agreement with sosui [20]. Protein building of the FFAR4 model was performed using modeller, version 9.25 [15, 16] and docking calculations were performed using autodock, version 4.2 [21, 22]. The FFAR4 protein model and PHS were imported into the docking software, in accordance with the developer’s instructions. The potential ligand binding sites of the FFAR4 model protein were calculated using autodock, version 4.2 [21, 22]. The hydrogen bonding energy, which is considered an important parameter for characterizing the interaction between GPCRs and their ligands [23, 24], was estimated in arbitrary units using molegro molecular viewer, version 7.0.0 (Molegro ApS, Aarhus, Denmark) [25].

### Expression vectors

The full‐length cDNA of human FFAR4/GPR120 (GenBank Accession No. BC101175) was amplified from human brain cDNA (Clontech, Mountain View, CA, USA) via a polymerase chain reaction and cloned into the p3XFLAG‐mycCMV vector (Sigma‐Aldrich, St Louis, MO, USA) to produce p3XFLAG‐h FFAR4 as described previously [7]. The alanine mutants of E249 (E249A) and R264 (R264A) of p3XFLAG‐h FFAR4 were obtained in accordance with manufacturer’s instructions for the PrimeSTAR® Mutagenesis Basal Kit (Clontech). The expression vector containing alkaline phosphatase (AP)‐tagged transforming growth factor‐α (TGFα) was prepared as described previously [26].

### Cell culture

293T cells were cultured as monolayers in Dulbecco's modified Eagle's medium (Sigma‐Aldrich) supplemented with 5% fetal bovine serum and penicillin/streptomycin at 37 °C in a humidified atmosphere of 5% CO_2_. Full details of the cells have been reported previously [27].

### TGFα shedding assay

The principles and a detailed method of the TGFα shedding assay have been described previously [7, 28, 29]. In brief, 293T cells were seeded in a six‐well plate. The next day, p3XFLAG‐h FFAR4, p3XFLAG‐h FFAR4 (E249A), p3XFLAG‐h FFAR4 (R264A) and AP‐TGFα were transfected into cells using polyethylenimine (Polysciences, Inc., Warrington, PA, USA) and cultured overnight. Cells were detached from the wells, re‐seeded in a 96‐well plate, and cultured for 30 min, after which ALA or a test substance was added. After being cultured for 1 h, AP activity in the supernatant and cells was determined using *p*‐nitrophenyl phosphate (Sigma‐Aldrich) as the substrate. Absorbance at 405 nm was measured using an iMark™ microplate reader (Bio‐Rad Laboratories Inc., Hercules, CA, USA). The data are presented as the AP activity in the supernatant as a percentage of the total AP activity. Figure 3 shows the AP activity of FFAR4 as 100% relative to E249A and R264A. Figure 4 presents the AP activity of PHS treatment as 100% relative to sphingosine (SPH) and dimethylsulfoxide treatment.

## Results and Discussion

### Docking simulation of ALA and PHS using a FFAR4 homology model

**Fig. 2:**
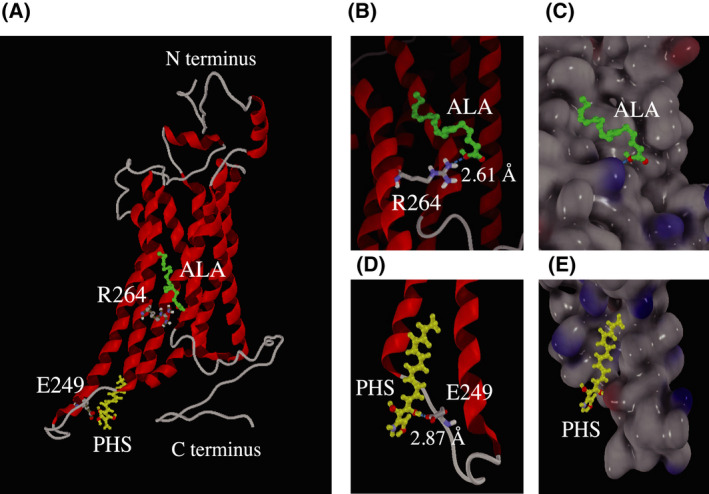
FFAR4 homology model docked with ALA and PHS. (A) The homology model of FFAR4 obtained in the present study. The docking simulation was performed using the model, and ALA (B, C) and PHS (D, E) were docked into the binding pocket of FFAR4 as described in the Materials and methods. Green, ALA; yellow, PHS; the predicted binding position according to the Molegro Molecular viewer (atoms, O; red, N; purple). The dashed blue lines indicated the distance between ligands (ALA and PHS) and residues (R264 and E249).

We previously identified PHS as a novel ligand of FFAR4 [7]. Many of the natural ligands found to date have carboxyl groups. However, PHS does not possess a carboxyl group, suggesting that its manner of interaction with FFAR4 may be significantly different from that of other natural ligands. To investigate this, we conducted docking simulation in a FFAR4 homology model that was developed on the basis of a protein model derived from the crystal structure. We searched GPCRs for crystallographic structures with high identity scores with respect to FFAR4 using the blast results. We selected the activated β_1_AR structure [19] as a template based on its sequence identity with FFAR4. β_1_AR possesses crucial basic amino acids for TM formation. The seven TM domains were predicted using sosui [20] (Fig. 1A). The alignment between β_1_AR and FFAR4 produced a high identity score (65.9%) and maximum query coverage (85%). There was 27.3% similarity between β_1_AR and FFAR4 (Fig. 1A). We used the crystal structure of β_1_AR [14] as a template to build a homology model of FFAR4 using modeller, version 9.25 [15, 16]. Finally, we successfully obtained a homology model of FFAR4 (Fig. 2A). The FFAR4 homology model was a typical GPCR possessing seven helical TM domains and a cytoplasmic C‐terminus [8]. ALA and PHS were then docked individually into the FFAR4 model using autodock, version 4.2 [21, 22] and viewed using molegro molecular viewer, version 7.0.0 [25] (Fig. 2B–E). The docking simulation of the FFAR4–ALA and FFAR4–PHS complexes revealed that the ligands were bound to the amino acids of FFAR4 via hydrogen bonds. We also built a predicted 3D structure of FFAR4 using the AlphaFold 2 database [30]. Although the 3D structure obtained was similar to the FFAR4 molecular model based on the crystal structure with β_1_AR, the 3D structure did not form any hydrogen bonds with natural ligand ALA in the docking simulation using autodock, version 4.2. Thus, we employed the FFAR4 homology model in this paper. The FFAR4–ALA model revealed possible hydrogen bonding between the oxygen of the carboxylate in ALA and R264 of FFAR4. The distance between the aforementioned oxygen and nitrogen of guanidine in R264 was 2.61 Å (Fig. 2B,C, Fig. S1C). The docking simulation of the FFAR4–PHS complex revealed possible hydrogen bonding between the oxygen of the hydroxy group at the 4‐position in PHS and the oxygen of the carboxylate of E249 of FFAR4, and the distance was 2.87 Å (Fig. 2D,E, Fig. S1C). PHS had two other weak hydrogen bonds in FFAR4 at S250 at distance of 3.25 Å, and at Q252 at distance of 3.35 Å, respectively (Fig. S1C). The distance of a hydrogen bond is typically 2.7–3.3 Å [31], and the distance of hydrogen bonds between E249 of FFAR4 and the oxygen of the hydroxy group at the C4‐position in PHS was 2.87 Å, representing a sufficient distance to permit hydrogen bond formation. Therefore, our docking simulations using a FFAR4 homology model suggested that PHS binds FFAR4 with a different molecular mechanism of binding from ALA.

### Significance of E249 and R264 in ALA‐ and PHS‐induced FFAR4 activation

**Fig. 3:**
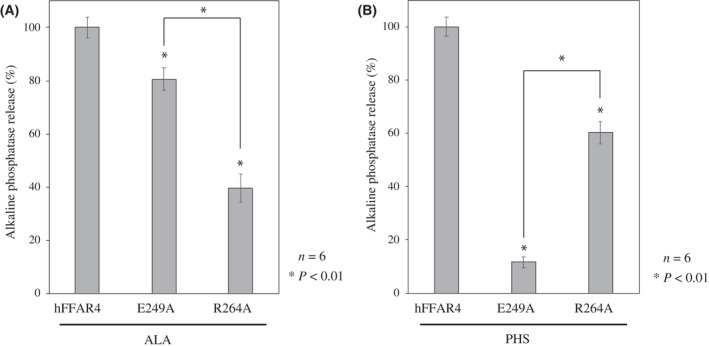
E249 mutation dramatically reduced PHS‐induced FFAR4 activation. We investigated whether ALA (A) and PHS (B) activate wild‐type FFAR4 and its mutants (E249A and R264A) using a TGFα shedding assay as described in the Materials and methods. ALA and PHS were dissolved in dimethylsulfoxide and added at a final concentration of 200 μm. The data show alkaline phosphatase activity of FFAR4 as 100% compared to that of E249A and R264A. Data were analyzed using Student's *t*‐test and are presented as the mean ± SD of six independent experiments; *P < 0.01 versus FFAR4.

We previously established a method for measuring the activity of FFAR4 using the TGFα shedding assay [7, 28]. Upon FFAR4 activation by ligands in the TGFα shedding assay, AP is released into the culture medium. Measuring this activity, we could quantify ligand‐induced FFAR4 activation. Using the method, we investigated the effects of alanine mutations (E249A and R264A) in FFAR4 on its activation by ALA and PHS. We found that the R264 mutation significantly decreased ALA‐induced FFAR4 activation, whereas the E249 mutation had less or only a modest effect on activation by ALA (Fig. 3A). Conversely, the E249A mutation severely decreased PHS‐induced FFAR4 activation (Fig. 3B). Interestingly, the effect of E249A was greater on PHS‐induced FFAR4 activation (Fig. 3B) than on ALA‐induced activation (Fig. 3A). Because our docking simulation identified possible hydrogen bonds between R264 and the carboxyl group of ALA (Fig. 2B) and between E249 and the hydroxy group at the C4‐position of PHS (Fig. 2D), these results support the accuracy of our model and clearly demonstrate that PHS interacts with FFAR4 with a different molecular mechanism of binding from ALA. On the other hand, the results for S250A and Q252A did not support a specific interaction with PHS (Fig. S1A and B). The S250 and Q252 might not be sufficiently close to form a stable bond with PHS.

To determine the precise effect of the mutations (R264A and E249A) on FFAR4 activation, we performed kinetic analysis and calculated *V*_max_ and *K*_m_ using the Lineweaver–Burk plot. As shown in Table 1, the *V*_max_ (%) values were 30.4 (FFAR4 to ALA), 31.7 (FFAR4 to PHS), 35.7 (E249A to ALA), 35.0 (E249A to PHS), 27.2 (R264A to ALA) and 27.3 (R264A to PHS). The *V*_max_ values of wild‐type and mutant FFAR4 with ALA and PHS were similar (Table 1). The *K*_m_ values (μm) were 11.8 (FFAR4 to ALA), 8.19 (FFAR4 to PHS), 39.3 (E249A to ALA), 80.6 (E249A to PHS), 63.1 (R264A to ALA) and 35.4 (R264A to PHS) (Table 1). Interestingly, the *K*_m_ values of FFAR4 mutant binding differed between ALA and PHS. Among the *K*_m_ values for ALA, the R264A mutant had the highest value (63.1 μm) (Table 1), indicating the lowest affinity for ALA. Because our docking simulation of the FFAR4–ALA complex indicated that R264 was important for binding with ALA, the increased *K*_m_ completely agreed with our model. By contrast, E249A had the highest *K*_m_ for PHS (80.6) (Table 1), indicating the lowest affinity for PHS. The E249 mutation dramatically reduced PHS‐induced FFAR4 activation (Fig. 3B) and our docking simulation of the FFAR4–PHS complex also revealed the importance of E249 for binding to PHS. These results strongly indicate that R264 and E249 are important for ligand binding with ALA and PHS, respectively. Therefore, we conclude that PHS activates FFAR4 through an interaction with E249, which is different from the position in ALA. Our results reveal that PHS acts on FFAR4 through a different molecular mechanism of binding from ALA.

**Table 1:** *V*_max_ and *K*_m_ values of wild‐type and mutant FFAR4 with ALA and PHS. To determine the precise effect of the mutation, kinetic analysis was performed using wild‐type and mutant (R264A and E249A) FFAR4, and *V*_max_ and *K*_m_ were calculated using the Lineweaver–Burk plot. *V*_max_ of FFAR4s were almost the same. *K*_m_ of E249A and R264A differed between ALA and PHS.

	Ligand	*V*_max_ (%)	*K*_m_ (μm)
hFFAR4	ALA	30.4	11.8
	PHS	31.7	8.19
E249A	ALA	35.7	39.3
	PHS	35.0	80.6
R264A	ALA	27.2	63.1
	PHS	27.3	35.4

### Significance of the hydroxy group at the C4‐position of PHS for binding to FFAR4

**Fig. 4:**
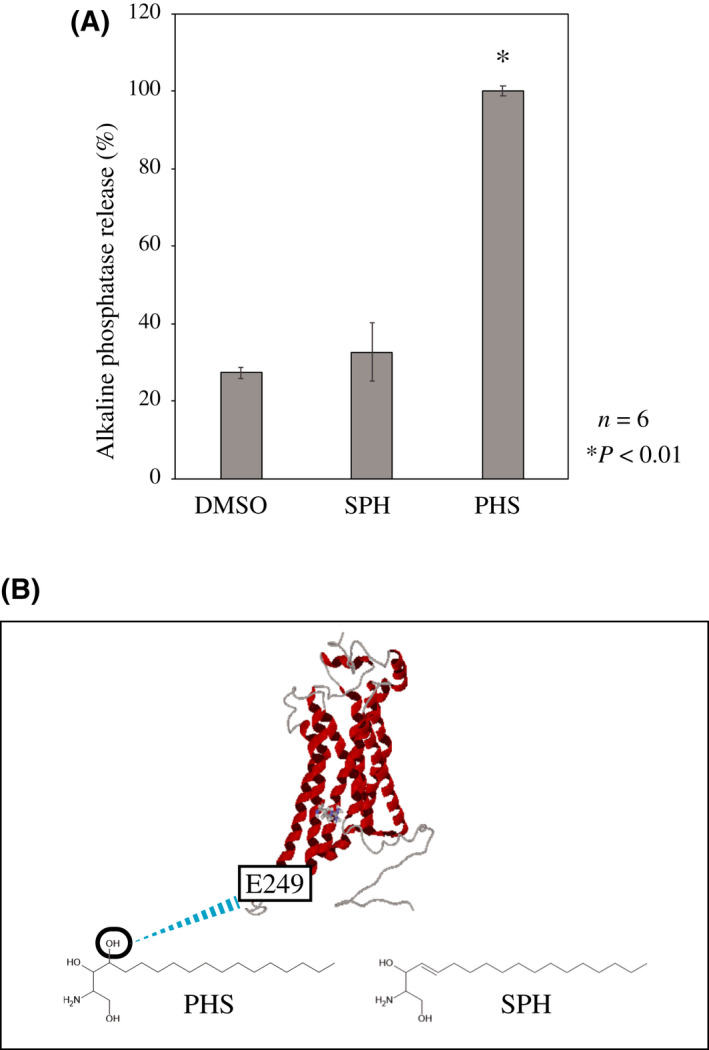
PHS activates FFAR4 through an interaction between the oxygen of the hydroxy group at the 4‐position and E249. (A) We investigated whether sphingoid bases (SPH and PHS) activate FFAR4 in a TGFα shedding assay as described in the Materials and methods. SPH and PHS were dissolved in dimethylsulfoxide and added at a final concentration of 25 μm. The data express the relative alkaline phosphatase activity of PHS treatment as 100% compared to that of SPH and vehicle (dimethylsulfoxide). Data were analyzed using Student's *t*‐test and are presented as the mean ± SD of six independent experiments; *P < 0.01 versus dimethylsulfoxide treatment. (B) There were possible hydrogen bonds between the oxygen of the hydroxy group at the C4‐position of PHS and the oxygen of the carboxylate at E249. SPH has a double bond at this position instead of a hydroxyl group.

SPH is a sphingoid base that is a constituent membrane sphingolipid in mammalian cells [32]. SPH and PHS displayed similar structures, having single long‐chain alkyl groups (C18) and an amino group at the 2‐position, although they differed at the C4‐position (hydroxy group versus double bond; Fig. 4B). Interestingly, we found that SPH hardly activated FFAR4 (Fig. 4A), indicating the significance of the hydroxyl group at the C4‐position in PHS for its interaction with FFAR4. According to our docking simulation, SPH could not form hydrogen bonds with E249 of FFAR4 because of the absence of a hydroxyl group at the C4‐position (data not shown). In addition to the lack of a hydroxyl group, SPH has a double bond at this position. This result also suggests that the oxygen of the hydroxy group at the C4‐position of PHS is important for the docking of E249 in FFAR4 (Fig. 4B).

## Conclusions

Our docking simulation of the FFAR4–PHS and FFAR4–ALA complexes revealed the possible existence of hydrogen bonds between E249 and PHS and between R264A and ALA. Interestingly, making a model and performing docking simulation with murine FFAR4, the same hydrogen bonds were observed between FFAR4, PHS and ALA (not shown). These bonds would be conserved in mammals. Measuring the ligand‐dependent activation of FFAR4 and performing kinetic analysis using E249A and R264A mutants, we revealed that PHS and ALA bind with E249 and R264, respectively, to activate FFAR4. Our results clearly demonstrate that PHS binds to FFAR4 through a different molecular mechanism of binding from many of the ligands having carboxyl groups. Additionally, the R264A mutation also showed a significant reduction in oleic acid‐induced FFAR4 activation (Fig. S2). The 3D structures of ALA and oleic acid are considered to be significantly different, suggesting that R264 is involved in the recognition of many FFAR4 ligands having carboxyl groups. Furthermore, we revealed that the oxygen of the hydroxy group at the C4‐position of PHS is critical for hydrogen bonding at E249. Interestingly, E249 is located in the cytosolic region (Fig. 1B, Fig. 2A). It is known that Sph generated on the cell surface rapidly translocates into the cell to be SPH 1‐phosphate [33, 34]. Accordingly, we assume that PHS could also diffusely penetrate cell and access E249 as well. Because the FFAR4 agonist has received increasing attention as attractive pharmaceutical agents for type 2 diabetes [35, 36], our findings may provide new and important insights into the development of novel FFAR4 ligands. PHS is found in the plasma membranes of yeast in large amounts and dietary PHS can be obtained by consuming bread and fermented foods. We previously reported that PHS improves impaired glucose tolerance in mice [37]. The docking simulation using a FFAR4 homology model, as established in the present study, may be useful for predicting the docking sites of ligands, and it is expected to become an important tool for investigating novel FFAR4 ligands.

## Conflict of interests

The authors declare that they have no conflicts of interest.

## Author contributions

**TN:** Investigation, writing – original draft. **MH:** Methodology. **S‐iK:** Methodology. **SH:** Resources; Methodology. **YH:** Validation; Supervision. **SM:** Conceptualization; Writing – review & editing; Supervision; Project administration.

## Supporting information


**Fig. S1.** The results for S250A and Q252A did not support a specific interaction with PHS. We investigated whether ALA (A) and PHS (B) activate wild‐type FFAR4 and its mutants (S250A and Q252A) using a TGFα shedding assay as described in the Materials and methods. ALA and PHS were dissolved in dimethylsulfoxide and added at a final concentration of 200 μm. The data show alkaline phosphatase activity of FFAR4 as 100% compared to that of S250A and Q252A. Data were analyzed using Student's *t*‐test and are presented as the mean ± SD of six independent experiments; *P < 0.01 versus FFAR4. (C) The length of hydrogen bonds between ligands and binding residues of GPR120 was calculated using molegro molecular viewer, version 7.0.0.

**Fig. S2.** R264 mutation dramatically reduced oleic acid‐induced FFAR4 activation. We investigated whether oleic acid activates wild‐type FFAR4 and its mutants (E249A, S250A, Q252A and R264A) using a TGFα shedding assay as described in the Materials and methods. Oleic acid was dissolved in dimethylsulfoxide and added at a final concentration of 200 μm. The data show alkaline phosphatase activity of FFAR4 as 100% compared to that of each mutant. Data were analyzed using Student's *t*‐test and are presented as the mean ± SD of six independent experiments; *P < 0.01 versus FFAR4. Although the 3D structures of ALA and oleic acid are different, almost similar results were obtained. This indicates that R264 plays an important role in the interaction with the carboxyl group of ligands.

## Data Availability

The data that support the findings of this study are provided in the figures and table of this article.
