# Supplementary figures and images for: The molecular mechanism of phytosphingosine binding to FFAR4/GPR120 differs from that of other fatty acids
Source: FEBS Open Bio. 2021 Oct 3;11(11):3081–9. doi: 10.1002/2211-5463.13301 (PMC8564095; doi:10.1002/2211-5463.13301)

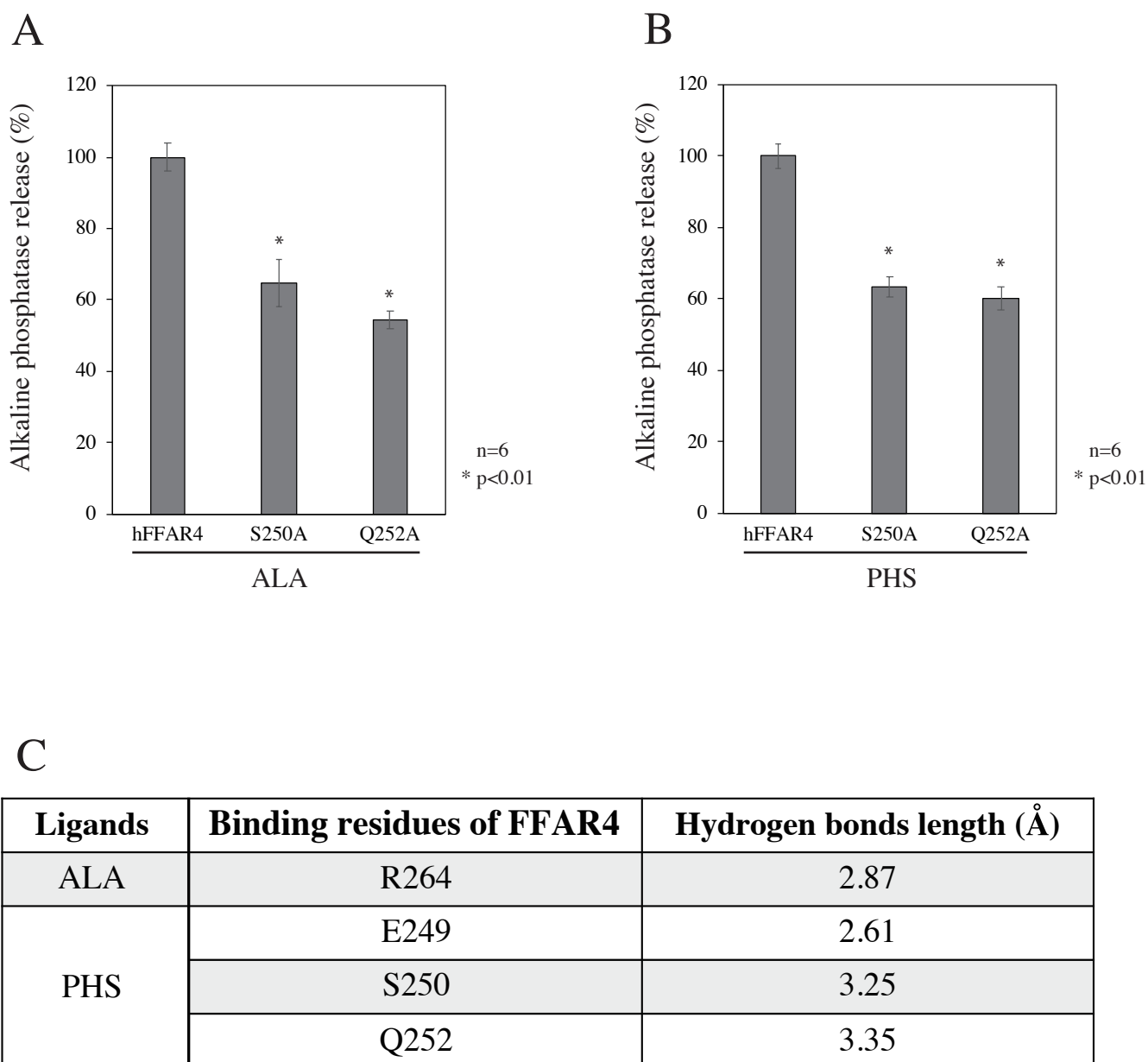

Fig. S1

Supplement: Supplementary file 1 — Fig. S1. The results for S250A and Q252A did not support a specific interaction with PHS. We investigated whether ALA (A) and PHS (B) activate wild‐type FFAR4 and its mutants (S250A and Q252A) using a TGFα shedding assay as described in the Materials and methods. ALA and PHS were dissolved in dimethylsulfoxide and added at a final concentration of 200 μm. The data show alkaline phosphatase activity of FFAR4 as 100% compared to that of S250A and Q252A. Data were analyzed using Student's t‐test and are presented as the mean ± SD of six independent experiments; *P < 0.01 versus FFAR4. (C) The length of hydrogen bonds between ligands and binding residues of GPR120 was calculated using molegro molecular viewer, version 7.0.0. [file FEB4-11-3081-s001.pdf]

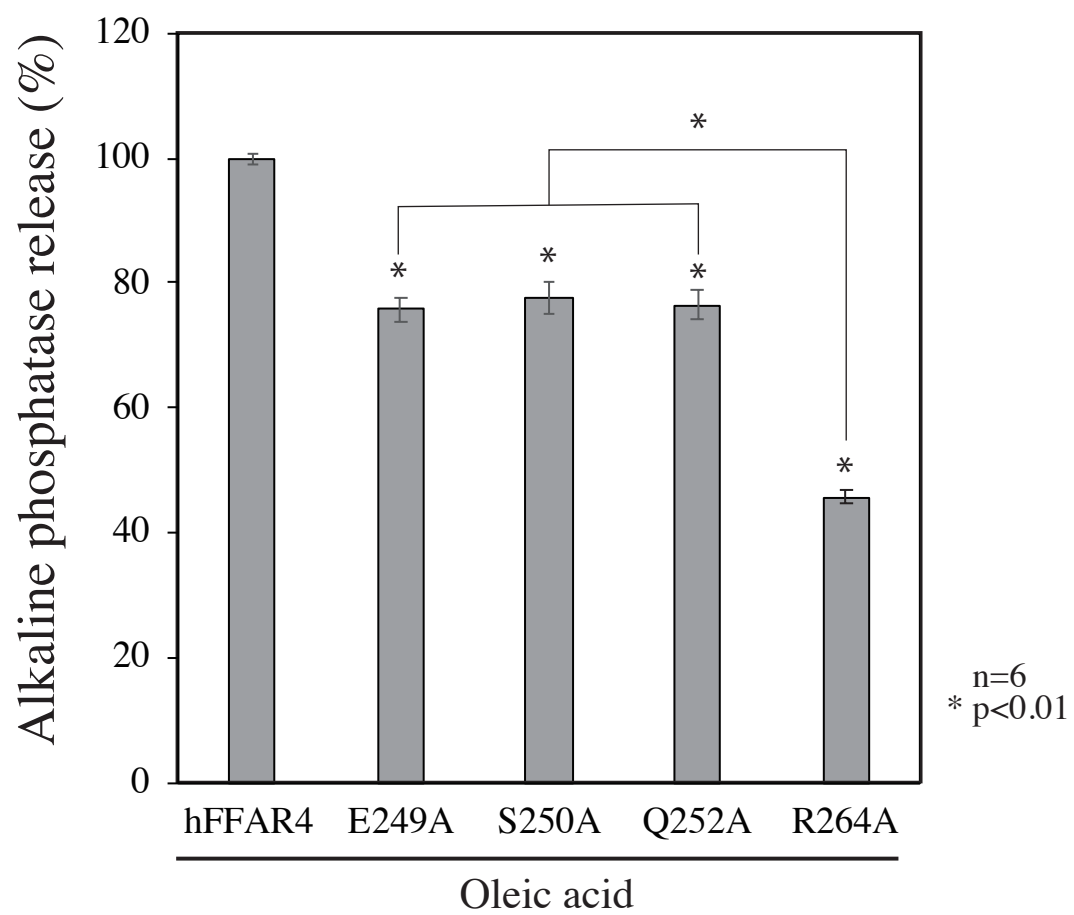

Fig. S2

Nagasawa T, et al.

Supplement: Supplementary file 2 — Fig. S2. R264 mutation dramatically reduced oleic acid‐induced FFAR4 activation. We investigated whether oleic acid activates wild‐type FFAR4 and its mutants (E249A, S250A, Q252A and R264A) using a TGFα shedding assay as described in the Materials and methods. Oleic acid was dissolved in dimethylsulfoxide and added at a final concentration of 200 μm. The data show alkaline phosphatase activity of FFAR4 as 100% compared to that of each mutant. Data were analyzed using Student's t‐test and are presented as the mean ± SD of six independent experiments; *P < 0.01 versus FFAR4. Although the 3D structures of ALA and oleic acid are different, almost similar results were obtained. This indicates that R264 plays an important role in the interaction with the carboxyl group of ligands. [file FEB4-11-3081-s002.pdf]
